# Emotions and Digital Well-Being: on Social Media’s Emotional Affordances

**DOI:** 10.1007/s13347-022-00530-6

**Published:** 2022-04-13

**Authors:** Steffen Steinert, Matthew James Dennis

**Affiliations:** 1 Department of Values, Technology and Innovation, Faculty of Technology, Policy and Management, Delft University of Technology, Delft, The Netherlands; 2 Department Philosophy and Ethics, Faculty of Industrial Engineering and Innovation Sciences, Eindhoven University of Technology, Eindhoven, The Netherlands

**Keywords:** Emotions, Feelings, Digital well-being, Affordances, Emotional affordances

## Abstract

Social media technologies (SMTs) are routinely identified as a strong and pervasive threat to digital well-being (DWB). Extended screen time sessions, chronic distractions via notifications, and fragmented workflows have all been blamed on how these technologies ruthlessly undermine our ability to exercise quintessential human faculties. One reason SMTs can do this is because they powerfully affect our emotions. Nevertheless, (1) how social media technology affects our emotional life and (2) how these emotions relate to our digital well-being remain unexplored. Remedying this is important because ethical insights into (1) and (2) open the possibility of designing for social media technologies in ways that actively reinforce our digital well-being. In this article, we examine the way social media technologies facilitate online emotions because of emotional affordances. This has important implications for evaluating the ethical implications of today’s social media platforms, as well as for how we design future ones.

## Introduction

Social media technologies (SMTs) have powerful effects on user’s emotions. Users often notice how their mood tracks the online messages and notifications they receive, or the updates and posts they view (Rost et al., 2016, pp. 1–3). These emotional effects are further magnified when users collectively generate, share, and comment on online content.^1^ Those who can vividly express their emotional states in a tweet or a post have the potential to act as lightning rods that channel the collective mood. Furthermore, today’s SMTs — used by Twitter, Tik Tok, Instagram, and Facebook — not only allow users to post content, but also allow them to “like” and comment on the posts of others. After posting, these comments can be commented upon (and “liked” again) ad infinitum. This results in a situation in which SMT’s users share their posts with a global audience of potentially millions of other users, while simultaneously receiving real-time feedback on the popularity (or otherwise) of these posts. No wonder this changes how users think and feel, both for better and worse.

Although it is common knowledge that SMTs affect the emotions of users, precisely how they do so is unclear. Our primary concern in the paper is to provide a better understanding of how the design of SMTs is connected to the emotional life of users. We believe that the most promising way to do this is via an account of the “affordances” that SMTs generate. As Michael Klenk puts it, affordances are “relational properties of objects that make a particular action more likely” (2021, p. 8).^2^ Affordances have much explanatory power when it comes to explaining how SMTs affect the emotions, behavior, and attention of users. We will introduce the notions of affordance and emotional affordance in more detail later in the paper.

In what follows, we will explore what we believe to be four key affordances in SMTs. These affordances allow users to (1) *express* themselves with personal updates, (2) *share* content when they repost, (3) encourage them to *consume* content, and (4) *evaluate* the content of others. We do not claim that this list is exclusive and that the affordances we pick out are especially connected to emotions.

All of these can be easily done with a click of a mouse, although how these affordances apply to specific users will vary. For example, the lives of those who experience some degree of marginalization in non-online life (minorities, the elderly, the disabled, etc.) can find the very same marginalization replicated (even magnified) when they use SMTs. While *self-expression* (1) is a key affordance of SMTs, marginalized individuals can find that this affordance is strictly curtailed for them or becomes something that actively detracts from their well-being. For instance, disabled users often report a sharp increase in trolling and online bullying when they post content that would be positively reacted to if posted by a similar able-bodied user. Nevertheless, if we bear these difference in mind, SMTs that possessed any one of the above (1–4) affordances would be likely to affect the emotional state of its users; one that possessed all four likely makes this all but inevitable.

We will show how affordances of SMTs affect the emotions of users in powerful ways, ways that impact — often negatively — on their *digital well-being*.^3^ Support for this claim comes from the fact that the affordances listed above (1–4) are invariably involved when users experience strong feelings, especially elation when their post is shared, liked, or goes viral (Burhan & Moradzadeh, 2020). Negative emotions, caused by the unkindness or malice of other users, have similarly been shown to lead to strong emotional reactions (Cocking and van den Hoven, 2019).

Furthermore, the affordances listed above have been chosen because they are *emotional affordances*; that is, they make it more likely for a user to experience, share, and express certain *emotions*.^4^ These affordances allow users to share even the most polarizing or contentious content, they increase our vulnerability to trolling or censorious evaluation, and they create a hypervisible public environment (with a permanent online footprint) in which this takes place. These factors mean that the emotional affordances of SMTs are inextricably attached to our digital well-being.

Our account also provides a useful vantagepoint to discern how tomorrow’s SMTs might be better aligned with digital well-being, which we address in the final sections. We believe that understanding how our digital well-being is affected by our emotions (Sect. 2) and how the emotional affordances of current SMTs affect our emotional life (Sect. 3), offer invaluable pointers for the designers of tomorrow’s SMTs. Although we do not write as designers ourselves, we believe that ethical insight in this area has clear practical application. Not only should such insight warn the SMT design community not to uncritically follow what we claim is an essentially “hedonic” conception of well-being, it allows us to address the tenacious problems that still beset the corporate design of SMTs. Understanding how the emotional affordances of SMTs affect our emotions is the first step to ground an improved set of design recommendations.

## Understanding the Influence of Emotions on Digital Well-Being

Before we focus on digital well-being and emotional affordances, it is useful to clarify what we mean by emotions. As occurrent mental states, emotions are intentional, which means they are directed at on object, like a person, or an event (Brady, 2013, 26 ff.). Another crucial feature of emotions is that they involve an evaluative appraisal, which means that something is evaluated in a particular way (Mulligan & Scherer, 2012). The aspect of appraisal links emotions to our concerns, which is why some philosophers, such as Robert Roberts (Roberts, 2003) propose to think about emotions as concern-based construals. This means that emotions are based on what people care about and on what they value. For instance, somebody is angry after an insult because they care about their reputation and the situation is viewed as a violation of this reputation. Because of concerns such as this, emotions make something appear in a certain light; they appraise something in a particular way. Another crucial feature of a lot of emotions (but not all) is that they involve action tendencies (Frijda, 1986), this means that emotions motivate or prepare behavioral responses. Emotions have a felt or experiential character. It usually feels a particular way to have emotions but emotions are not the same as feelings. Many feelings are non-emotional, like hunger or arousal, and two or more emotions may involve feelings of the same bodily changes (Brady, 2013, 40 ff.).

It is important to distinguish emotions from other affective states, such as moods. Emotions and moods should not be conflated, despite the fact that they have some common features, like being felt. Emotions are usually short-lived affective experiences. Another crucial difference is that moods are not directed at concrete objects. Rather, the object of a mood, like depression, is the appraisal of the total environment in a particular evaluative light (Mitchell, 2021).

Recently, ethicists of technology have devoted much attention to the value of emotions in how we design online technologies (Marin & Roeser 2020; Steinert, 2020), as well as to the importance of designing for DWB specifically (Burr et al., 2020; Burr & Floridi, 2020; Brey et al., 2012; van de Poel, 2012). Thinking about how the good life and the emotions are connected has a long and distinguished philosophical heritage, one which is useful to bear in mind when discussing this topic. Philosophers have claimed that emotions are an essential aspect of living well for a very long time. Both *hedonistic* accounts (which focus on pleasure) and *eudaimonic* accounts (which focus on human flourishing) typically agree that the right emotional state is an important part of human well-being. Nevertheless, philosophers in the hedonic tradition (from Epicurus to J.S. Mill) view the way the emotions affect well-being in strikingly different ways to those in the eudaimonic tradition (from Aristotle to modern virtue ethicists).^5^

Bringing eudaimonic well-being into the debates of digital well-being broadens the scope of the debates of digital well-being, which currently tends to emphasize hedonic conceptions.^6^ As we show below, concentrating on eudaimonic well-being has advantage because eudaimonic theories have a much broader conception of the role that emotions play in well-being, viewing a good life as one that is open to positive *and* negative emotions. Furthermore, most often, the design of today’s SMTs favor pleasure, implicitly aligning with a predominantly hedonic conception of well-being.^7^ We will argue that digital well-being can be advanced if the design of SMTs focus on eudaimonic well-being. The aim of the following sections, then, it to show how these differing conceptions to well-being affect how we should think about the role of emotion in digital well-being.

### Hedonic Conceptions of (Digital) Well-Being

Hedonists claim that well-being should be understood in terms of the attainment of pleasure and the avoidance of pain (Kahneman et al., 1999; Ryan & Deci, 2001).^8^ This bears on how hedonists view emotions. On this conception, a life that goes well is one in which positive emotions dominating negative ones because positive emotions are caused by — while also generating — pleasure. For this reason, hedonic accounts emphasize the importance of positive emotions (joy, love, cheerfulness), and claim that negative emotions (sorrow, fear, disappointment) should be avoided.^9^

Hedonic accounts of WB tend to focus on emotions because of the “valence” or “hedonic tone” of emotions (Frijda, 1986; Mulligan & Scherer, 2012). This means hedonic accounts assume a polarity of emotions such that most emotions are categorized in a binary fashion as either pleasant or unpleasant, positive or negative. Positive emotions, such as joy, are typically pleasant and experienced as positive. In contrast, negative emotions, like fear and sadness, are usually more salient in negativity. However, some research indicates that emotions contain both positivity and negativity (An et al., 2017). So, because hedonic well-being is characterized by the attainment of pleasure and the avoidance of pain, and some emotions are pleasant or unpleasant, positive emotions are a vital part of WB on the hedonic account.

Nevertheless, the hedonic conception of WB has problems, and many philosophers ultimately find it unsatisfying. For instance, it seems that it cannot fully account for many of the essential things that seem to contribute to a life going well. To put the point straightforwardly, it is hard to dispute that human beings care about many other things than pursuing pleasure.^10^ Furthermore, a lot of meaningful goals that contribute to WB involve a lot of displeasure. For instance, increasing one’s bodily fitness, or pursuing a demanding career, include lots of things that are not pleasurable (and can be highly unpleasant), but are pursued by many people nonetheless. Also, consider that many things that are pleasurable and desirable from the perspective of the person but could not be said to promote WB. Hard drugs may bring intense pleasure, for instance, but few would argue that they contribute to WB. To take another example, some pursuits may contribute to a lesser degree to WB than pursuits of with the same hedonic quality. Watching a Netflix show and participating in a neighborhood committee, say, may generate the same amount of pleasure, but the latter, according to Susan Wolf, has much more potential for meaningfulness (2016, p. 58). Many will share Wolf’s intuition.

So how does this apply to social media? Currently most SMTs have a hedonic focus, favoring hedonic WB over eudaimonic WB. Receiving recognition and approval online, for instance, in the form of “likes,” gives people a positive feeling of elation and satisfaction.^11^ Moreover, excessive social media use can be at least partly explained by people’s emotional and hedonic relation to the technology (Twenge, 2018). As we will show later, SMT companies actively encourage this because it increases traffic on their platforms. By designing their platforms in ways that facilitate the experience of short-term pleasure, these companies hope to become more profitable (Zuboff, 2019). In fact, some have argued that it is an essential part of their business model (Bhargava & Velasquez, 2021; Harris, 2018).

Nevertheless, despite the powerful influence of SMTs, users still retain agency. For instance, users often employ social media to control their affective states to enhance their (hedonic) DWB. This phenomenon could be called “digital emotion regulation” (Wadley et al., 2020). The posting of personal news or achievements, for example, can precipitate positive emotions, such as pride or self-satisfaction. Marcus Gilroy-Ware (2017) makes a compelling case that a lot of people use SMTs for pleasure-seeking consumption to cope with the strains of life in advanced capitalistic societies.^12^ For these reasons, we may want to resist a simplistic narrative that focuses only on how social media manipulates people’s behavior. Rather, the story needs to include an account of how SMTs reinforce the hedonic model by defining and facilitating how users seek a certain kind of pleasure.

### Eudaimonic Conceptions of (Digital) Well-Being

While hedonistic conceptions of WB focus on an important aspect of social media use, we contend that these conceptions do not tell the whole story. This is because, as we have seen, there are reasons to doubt that real WB involves a constant stream of positive emotions or feeling good all the time. In fact, as mentioned above, the *eudaimonic* approach to WB suggests that negative emotions are necessary for a life that flourishes. As we noted above, Wolf (2016) suggests that this includes things like self-realization and living a purposeful or meaningful life.^13^ On this account, there is something missing from hedonistic approaches. In the words of Thomas Scanlon, “well-being depends to a large extent on a person’s degree of success in achieving his or her main ends in life […]” (1998, p. 124). More recently, Valerie Tiberius (2015) has defended a value-based approach to WB. According to her, a person’s life goes well to the extent that what the person values is pursued, fulfilled, or realized. All these thinkers agree that we live well by pursuing the goals we care about, not by merely enjoying one pleasurable experience after another.

This indicates that there is a rather more complicated relationship between emotions and WB than the hedonists suggest. Negative emotions may have to be endured in order for us to achieve the goals that will lead to true life satisfaction. Moreover, emotions can actively promote the pursuit of meaningful ends. Recall that emotions are linked to our concerns and that they appraise objects and events according to these concerns. Emotions are “relevance detectors” (Frijda, 1986; Mulligan & Scherer, 2012) and concern-based construals (Roberts, 2003). This means that emotions are able to alert us to things that have implications for our concerns and values; emotions allow us to identify and pursue meaningful goals and identify obstacles to the realization to these meaningful goals.

Nevertheless, the role of emotions for eudaimonic WB goes beyond signaling that a thing has implications for meaningful goals. Michael Brady (2013, 2016), for instance, makes a persuasive case for why emotions capture and consume our attention. The pursuit and realization of meaningful goals requires both self-understanding, he suggests, as well as an understanding of these goals (2016, 2013). Emotions can facilitate this understanding. Because they hold our attention, emotions put us in a privileged epistemic position to evaluate whether something really will contribute to our long-term goals. The understanding that something has implications for values and meaningful goals, and an understanding of these goals and values itself, is necessary for pursuing them. It is because emotions facilitate this understanding that emotions contribute to eudaimonic WB.^14^

For the eudaimonist, the hedonic approach to WB ignores the central role of negative emotions for human flourishing (Kashdan & Biswas-Diener, 2015; Tappolet et al., 2018). The eudaimonic approach recognizes negative and mixed emotions as important for WB. Because emotions signal that something has implications for our concerns, negative emotions can signal that something has implications for our flourishing, even if it signals that we should avoid this thing. For instance, feeling dissatisfaction may signal that something in your life is amiss. This could motivate people to take stock of their life and investigate the reason for the dissatisfaction, thereby contributing to flourishing and fulfillment. Similarly, guilt can facilitate virtuous conduct. Furthermore, researchers (Berrios et al., 2018) have shown that mixed emotions — experiencing positive and negative emotions at the same time — can be positively correlated with eudaimonic WB. This is because mixed emotions are associated with complex goals that often contain conflicting elements.

In addition to this, the design of SMTs with regard to emotions may even conspire against eudaimonic WB in ways that hinder human flourishing. Take online outrage, for example. Above, we noted that pursuing meaningful goals is a vital component of eudaimonic WB. Although online outrage can rally group members to pursue a goal that contributes to collective WB, William Brady and Molly Crockett (2019) describe how online outrage can backfire for certain groups. One function of online outrage is to signal to members of the group that a goal is worth pursuing collectively. Nevertheless, because the bar for expressing outrage on social media is very low, a constant flurry of outrage can often make it harder for a group to discern which goal is worth pursuing. As a consequence, the group may fail to detect the best cause for action that will lead to the WB of the group as a whole. In a similar manner, online outrage may provoke a group to pursue a goal that seems to serve collective well-being, but is actually detrimental to it.^15^

To summarize, there is a crucial link between emotions and WB, and social media has the power to shape and influence the emotions of users. Nevertheless, WB need not be conflated with hedonic WB, with its focus on positive emotions and pleasure. Eudaimonic WB is more relevant as it is not as limited as hedonic WB; it can even encompass the hedonic dimension. For these reasons, we propose that SMT should be designed with eudaimonic DWB in mind.^16^ Focusing on eudaimonic digital WB can guide to think about how to use digital technology to achieve a meaningful life and flourish as a human being.

Unfortunately, SMTs often seem to be designed with a focus on hedonic WB and pleasure. As a first step to illuminate how emotions are linked to eudaimonic DWB, it is necessary to understand how the design features of SMTs are linked to particular emotional affordances. It is to emotional affordances that we will now turn.

## Affordances and Emotional Affordances

The notion of emotional affordance is an extension of the idea of affordance, which was developed by psychologist James Gibson. Generally speaking, affordances are action possibilities of the environment that are available to an organism. In Gibson’s own words, affordances are what the environment “offers the animal, what it provides or furnishes, either for good or ill” (Gibson, 1979, p. 127). So, the concept of affordance describes the relationship between the organism and the environment.^17^ How the organism perceives the environment, or objects in the environment, depends on the capabilities of the organism and its needs. For instance, a cave can afford shelter from the elements or hiding from a pursuing predator. Furthermore, a stick can afford protection from an attacker or leaning on when one is injured. Because affordances are relative to individual needs and capabilities, the same environment or object can afford different things to different organisms or different things to the same organism at different times.^18^

Affordances are not limited to items of the natural world, like sticks, caves, and rocks. The social world is also full of affordances. The outstretched hand affords shaking it and being waved at by an acquaintance may afford waving back. Technical artifacts have affordances too. Oftentimes, designers design technical artifacts with affordances in mind because the designers want the user to handle the item in a particular way. Don Norman famously introduced the idea of affordances in the field of human technology interaction. According to Norman, affordances are “[…] perceived and actual properties of the thing, primarily those fundamental properties that determine just how the thing could possibly be used” (Norman, 1988, p. 9).^19^ Consider, as example, that in virtue of their features, a door knob affords grasping and the computer mouse or the trackpad both afford a particular way of handling. However, sometimes technical artifacts have affordances that the designer did not intend and that only become relevant in specific use situations. For example, in some situations, a paper weight may afford using it as a door stopper. Similarly, a cup can be used for drinking and to extinguish a flame if necessary.^20^ Compared to artifacts like cups and paper weights, software is more versatile and malleable. This sets software, and by extension SMT, apart because it can be deployed in many different contexts and has a plethora of different use cases.

Affordances are not limited to possibilities of bodily actions and the idea can be extended to include emotions. To give a better idea about what emotional affordances are, consider that social interaction is characterized by inter-body dynamics, like facial expressions and gestures. Bodily dynamics are also important for picking up on the emotions of others and expressing own emotions.^21^ The bodily movement of others can afford particular reactions, like trying to comfort them. It can also afford emotional experience similar to the one expressed by the other person. This phenomenon is called emotion contagion (Hatfield et al., 1994). As we will address later, even online emotion contagion is possible.

So far, we have characterized e*motional affordance* as relating to elements in the environment that provide opportunity for emotional reaction. However, we would like to stress that emotional affordances go beyond the capacity to elicit an emotional reaction. Emotional affordances also provide opportunities to enact emotional experiences. As Christoph Bareither (Bareither, 2019) notices in a discussion of emotional affordances, emotions are not discrete and isolated instances but are complex processes that people actively shape. Hence, emotional affordances encompass the “[…] capacities to enable, prompt and restrict the enactment of particular emotional experiences” (Bareither, 2019, p. 15).

Emotions and affordances play a huge role in design (Norman, 2005) and emotional affordances are not limited to social media technology. Given the importance of emotional affordances, it is perhaps unsurprising that designers have discovered that emotional affordances play a crucial role in the human-technology interaction.^22^ For instance, emotional affordances have been used to facilitate human–robot interaction (Vallverdú & Trovato, 2016) and to develop online learning environments that foster emotional learning (Park & Lim, 2019). We would like to reiterate that our focus here is on affordances and emotions but we think our account can cover SMT affordances and other affective states, like moods.

Because affordances are relational phenomena (Chemero, 2003), they depend on properties of the technologies and features of the agent interacting with it. Features of the agent include their cognitive and bodily constitution but also cultural knowledge and social norms. For instance, the world affords different things depending on whether you are a child or an adult. Similarly, as Michael Hammond (Hammond, 2010) observes, perception of affordances is influenced by past experiences and conventions. Affordances depend on cultural and social background. An outstretched hand, for instance, affords grabbing and shaking it only in groups where this form of greeting is a social norm.

The features of the individual that uses the technology matter but the properties of technology matter as well because affordances are relational. So, we should acknowledge that SMT is a heterogenous group of technology. Different SMT can have different kinds of affordances. Furthermore, social media content can be delivered via a variety of platforms, like webpages and smart speakers, which can add affordances and foreclose other. For instance, a social media message.

An upshot of the dependency of affordances on properties of the individual and properties of the technology is that there will be individual and cultural variety of emotional affordances that SMT generates. Members of different social groups may perceive different emotional affordances. Consequently, when we talk about emotional affordances of SMT below, we are not claiming that they are fixed or that some features of SMTs will definitely cause individuals to behave a certain way. Despite this potential variety, it is nevertheless possible to distinguish crucial clusters of emotional affordances of SMT.

We will not go into detail here but we think that different bodies of literature can yield fruitful insights into emotional affordances. For instance, to further explore the issue of emotional affordances, one could enlist post-phenomenology and STS. Post-phenomenology, with its focus on mediation, can provide helpful insights into how technology shapes our experiences (Verbeek, 2016). This could be extended to address how technology shapes emotional experiences. There is interest in emotion from STS scholars who have looked into network affects. These are affective experiences related to online technology and social media (Hillis et al., 2015). Authors in digital STS research have further developed the actor-network theory of Bruno Latour to include so-called emotive actants (Stark, 2019).

## On the Emotional Affordances of Social Media Technologies (SMTs)

It is widely acknowledged that SMTs can influence an individual’s affective state. We say affective state here because we want to include the possibility that SMTs can have an effect on different affective states, like emotions and moods. Using SMT may cause an emotional reaction but it may also maintain or cause a particular mood. This could happen via an affective shift where one affective state shifts to another (Mitchell, 2021). It is possible to experience different kinds of affective shifts. One can change from one emotional state to another. An affective shift can also occur from emotion to mood, and conversely, from mood to emotion. For instance, reading an amusing social media post could diffuse into the mood of cheerfulness. Or, another social media post could shift an extant mood of anxiety into the emotion of fear. Depending on which affective state is involved and because different affective states have different durations, social medias’ effect can be short or long term. In what follows we will concentrate on emotions but this does not exclude the possibility that this has implications for other affective states, like moods.

Although SMTs influence users’ affective states, there has been little work done on how these effects are magnified by the hyperconnected contexts SMTs create. This is odd because the hyperconnected nature of SMTs is a key part of understanding why these technologies influence our emotional states so radically. SMTs connect us, but their emotional affordances involve network effects that compound the emotional effect of every post encountered. Grounded in the literature on affordances and emotions, we define emotional affordances as the *relational properties of SMTs that are likely to induce an emotional state or emotion-related behavior*, like the expression of a certain emotion or the reaction to the expressed emotional state of others.

SMTs have a number of emotional affordances, all of which have the potential to affect digital well-being.^23^ In what follows, we explore four key emotional affordances of social media technologies: (1) expressible, (2) shareable, (3) consumable, and (4) evaluable. Each of these emotional affordances generate emotions and behaviour related to emotions that have a profound effect on digital well-being but do not contribute to eudaimonic well-being.

### Expressible

SMTs have a range of emotional affordances that make it quick, simple, and easy to express emotion, which makes expressing emotion more likely. To borrow a term from Jenny Davis's and James Chouinard’s framework mentioned above (2016), these design features “encourage” users to express themselves. Indeed, some of the design choices of the platform seem purposely intended to *afford* emotional reactions and expressions. For example, in 2016 Facebook expanded its infamous like-button. From this point onward, users could use a button with various emojis to express their love, laughter, sadness, and anger about a post. Currently there is even a “wow” emoji that lumps together emotions ranging from astonishment to awe.^24^ Providing a limited option space of emotions that are available for expression is in itself interesting from the affordance perspective. Limiting the number of emotions that can be expressed affords a decision of which alternative best fits the emotional state. Oftentimes, however, emotions are not clear cut and do not fit neatly into a certain category.^25^ Another important way that SMTs affords the expression of emotional reaction is that the threshold to express an emotion is very low. Part of the explanation is that most social media platforms are practically anonymous. So, to give vent to one’s feelings is only matter of reaching towards one’s trackpad or mobile device, particularly when it comes to the expression of negative emotions, like outrage (Brady & Crockett, 2019; Crockett, 2017).

Not only do the emotional affordances of SMTs make it easy to express emotion, these technologies feed their users a regular flow of highly emotive content to which many respond. This content, including content that depicts the emotions expressed by others, offer countless opportunities for emotional reactions. Because SMTs make expression so easy, users are often inclined to express their emotional reactions in response to an emotional expression by others (as we will also discuss in the next section). This can lead to virtuous or vicious cycles of highly emotional responses, quickly followed by a highly emotional counter-responses.

In addition to this, because much online communication is text based and words are limited, it affords the expression of emotions that lend themselves to brief textual expression.^26^ Put differently, the design “demands” (Davis & Chouinard, 2016) that the user is brief and they “discourage” both expressing complex emotions and the complex expression of emotions. Complex or ambivalent emotions, like embarrassment or remorse, are harder to fit into the mold of word limits and image-based communication of SMT.^27^ Doing justice to these complex emotions requires elaborate expressions that can cover the nuances and particulars of the mental states involved. The affordances of SMT, and their focus on easily expressed emotions, thus militate against eudaimonic WB because eudaimonic WB requires that we properly deliberate about our emotions and do not neglect complex or ambivalent ones.

Another implication of the emotional affordances of social media for eudaimonic WB has to do with the fact that social media encourages the presentation of a warped and somewhat unauthentic self-image, including the expression of unauthentic emotions, partly because SMT creates norms and expectations about what emotions to express. For instance, on platforms like Facebook and Instagram, there seems to be a norm to express positive emotions (Waterloo et al., 2018). The bias towards posting positive emotions may further exacerbate the social pressure not to feel negative, which is associated with a negative self-concept (Dejonckheere & Bastian, 2020). Indeed, recently researchers (Bailey et al., 2020) found that posting authentically on social media, instead of in an idealized fashion, leads to greater well-being and life satisfaction.

### Shareable

The design features of SMTs also afford sharing of another’s emotional content with ease. When it comes to sharing, both emotional affordances and behavioral affordances work together. For instance, the posts of other users can create an emotional reaction in us, particularly when they include emotive content. Because emotions motivate user to act, these emotionally evocative posts afford not only an emotional reaction but also actions, like the inclination to share or to instantly respond. SMTs have affordances that makes sharing easy. The design features of SMTs “encourage” sharing, for instance, with prominently displayed buttons. Similarly, push notifications afford sharing because they alert the user to potentially shareable content (or to others sharing content).

The way social media can afford both expression and sharing of emotions is important because users can affect other users with their emotions. This is known as emotional contagion. Because social media affords emotional sharing, and because the reach of social media is huge, it should come as no surprise that there is *digital* emotion contagion. Digital emotion contagion (Goldenberg & Gross, 2020) means that the emotion of someone perceiving an emotional expression online becomes more like the emotions of the user that posted emotional content.

We should consider “shareability” to be one of the most powerful emotional affordances that SMTs offer because emotional content spreads more quickly and easily than non-emotional content. Researchers found that when social media messages include emotional-moral terms, their spread increases substantially (Brady et al., 2017). Furthermore, users are more inclined to share content that triggers moral emotions, like outrage (Crockett, 2017, p. 20), and anger spreads faster and reaches more users than other emotions (Fan et al., 2020).^28^

The combined effect of the affordances of expresssibility and shareability lead to constant exposure of emotional content online. This may have a negative impact on eudaimonic DWB. For instance, on the group or societal level emotional contagion could lead to an emotional atmosphere (Steinert, 2021) dominated by negative emotions. Because negative emotions motivate to avoid a perceived threat, this could motivate people to concentrate on less meaningful goals or goals that are opposed to their flourishing. This is likely not limited to user’s time social media because online and offline life cannot be neatly divided. Also, as current incidences, like the storm on the US Capitol, attest, people pursue their goals both online and offline.

It needs to be noted that sharing of emotions online can positively affect the sharer’s well-being, especially when there is a form of intimacy (Lomanowska & Guitton, 2016). For instance, emotional self-disclosure could lead to social support and stronger social connection, which are essential aspects of human flourishing. Nevertheless, because SMTs are geared towards simple sharing with one click, and because they limit responses to a number of words, this discourages a deeper and more meaningful response, and deep engagement with the emotions of others.

### Consumable

The emotional affordances of SMTs also facilitate the quick, easy, and convenient consumption of emotional content. Consider push notifications. These electronic reminders enable consumption of content by engaging the attention of users, even when these users are not using the device. Push notifications not only push content, but also pull the user out of whatever they are doing into the realm of social media. In other words, push notifications “request” (Davis & Chouinard, 2016) that users consume content.

Another design feature that lends itself to easy consumption is the default option to stay logged in. Here, the user is “encouraged” to stay logged in, which makes easier the consumption of the continual flow of online content. This is compounded by another ubiquitous design feature of today’s SMTs. These technologies provide visible (and prominent) metrics that are often regarded as central to user experience. Seeing the number of “likes,” “shares,” and “upvotes” creates another pull towards consumption of this content because users are typically interested in what other users find interesting. This can lead to a so-called “follow-the-crowd” phenomenon,^29^ where content that has received a lot of attention receives ever more attention. This, in turn, means that this content is taken to be more credible or valuable by other users, which enhances shareability.

The combined power of affordances related to expression, sharing, and consumption work together to create a cycle where emotional content is expressed, consumed, then shared to be consumed by others. This can have implications for WB (see fn. 3 above). Take social comparison as an example here. For a targeted group of users, platforms like Instagram encourage uploading photos in a particular style or editing photos so that they comply with the dominant aesthetic language, encouraging users to present themselves in ways that the algorithm considers to be best.^30^ On social media, users often encounter curated presentations of the life of other users, such as a constant stream of photographs depicting glamourous holidays, muscular bodies, and active social lives.^31^ The comparison and mismatch between their own life and what they see online can lead to negative emotions. Studies indicate that being exposed to posts on social media like Facebook can lead to envy (Tandoc et al., 2015), depression (Steers et al., 2014), and has been found to generally reduce subjective well-being (Kross et al., 2013; Verduyn et al., 2015). Self-acceptance, which includes awareness and acceptance of strengths and weaknesses, is important for eudaimonic WB (Ryff & Singer, 2008). The constant comparisons facilitated by the affordances of SMT, and the negative emotions related to self-image that go along with it, do not support self-awareness and self-acceptance. This can stand in the way of self-realization and personal growth, both relevant for eudaimonic WB.^32^ To summarize, for many users the negative emotions of social comparison do not help them in their pursuit of their values but merely paralyze them.

### Evaluable

The design of today’s SMTs also create emotional affordances that are related to evaluation. Strategically placed “like” buttons, thumbs-up buttons, heart buttons, and emojis invite users to constantly evaluate content, in real-time. This can take the form of retweeting, liking, or upvoting.

People strongly respond to the number of “likes” they receive. Studies point out that receiving online recognition in the form of likes and other forms of approval engages the reward network of the brain. Rewarding the brain in this way, gives users a dopamine-laden high, which psychologists relate to positive feelings of elation and happiness. In a study where they scanned teenagers’ brains, Lauren Sherman and colleagues (Sherman et al., 2016) found that seeing a lot of likes for their own photos or photos of peers engages precisely the same circuits as eating chocolate.

The implementation of these addictive making evaluation mechanisms only underscores the hedonic outlook of SMTs. The above-mentioned metrics and follow-the-crowd phenomena (Sect. 4.3) only exacerbate the issue insofar as they also afford evaluation. Users engage more with the posts of other users when these posts have already gathered a lot of likes. For instance, in the study by Sherman and colleagues that we mentioned above, adolescents are more likely to endorse pictures that have many likes, compared to pictures that have only a few likes (Sherman et al., 2016). This behavior can be explained with processes like social influence bias, which can lead to herding effects (Muchnik et al., 2013), or, take what psychologists call “mirroring” (Chartrand & Bargh, 1999), that describes the process of unconsciously imitating other people. So, it is likely that knowing the emotions that others feel towards a certain post will affect how people feel towards it. However, this does not exclude other possibilities of why users engage with posts that already received a lot of attention from other people. For instance, the topic of the post may simply be of interest and is related to one’s concerns.

Here again, we witness the combined power of emotional affordances: sharing leads to more users viewing the content concerned, which in turn leads to more evaluations, which in turn leads to more views from others. All this is compounded by the effects of the algorithms that are routinely upvote emotional content.

The evaluative aspects of social media have multiple implications for DWB. In the clearest sense, being evaluated negatively or positively (or not receiving evaluation at all) can have hedonic implications. For instance, receiving negative evaluations or no evaluation at all, such as when a post is ignored and not shared by others, can leave one with negative emotions like shame, regret, or anger.

More importantly, affordances related to evaluation have implications for eudaimonic WB. For instance, being acknowledged online can be a cause of pride and can contribute to a positive self-image, which is an important aspect of eudaimonic WB. Furthermore, the praise one gets on social media can motivate to continue to pursue one’s meaningful life goals. In contrast, being very much attached to the opinions and acknowledgment of others can cause feelings of not being good enough when the acknowledgement falters. Ultimately, this could motivate to give up on some goal or activity of self-realization.

Another eudaimonic implication of the emotional affordances related to evaluations is that these affordances encourage a purely quantitative engagement with content. That is, it is all about the numbers of likes, retweet, and shares (see also Sect. 4.2). This does not contribute to deeper engagement and evaluation of emotion content. Nevertheless, a more meaningful engagement with the emotions of others would make a more valuable contribution to eudaimonic well-being.

## Conclusion

In this article we have made the case that it is crucial to focus on how SMTs impact on the emotional life of users. We showed that emotions are intrinsically linked to the WB of users, and that the emotional affordances of SMTs mean that our digital well-being is especially vulnerable if these affordances are exploited. Understanding the effects of emotional affordances is a first step in advancing theoretical understanding of how the design of SMTs is linked to the emotional life of users and to their digital well-being. We hope this closes a gap in the literature. Much ethical discussion of social media revolves around their behavioral affordances, but the designers of tomorrow’s SMTs need to appreciate the power of emotional affordances, if they are to design in ways that are compatible with digital well-being. If emotional affordances of SMTs still remain largely unnoticed, designers are not motivated to pay attention to them. To ameliorate this, we have explored four emotional affordances of social media (Sects. 4.1, 4.2, 4.3, and 4.4). All these affordances have a powerful effect on the hedonic and eudaimonic well-being of users alike. However, while the design of SMTs is guided by a hedonic conception of WB, the importance of negative emotions in digital well-being will continue to be neglected. Focusing on eudaimonic digital well-being has advantages because such a focus enables us to give a better account of the relationship between social media affordances, emotions, and well-being. This is a tough challenge for the designers of SMTs, but it also seems that it would increase the ability of SMT platforms to retain their users. We hope that this theoretical contribution can facilitate a design of future SMTs that focuses on the eudaimonic digital well-being of users in ways that pay attention to the power of emotional affordances.

## Data Availability

Not applicable.

## Footnotes

1 The first quarter of 2020 provided numerous instances of this. Outrage at US policing practices (or ambivalence), sharing vital epidemiological knowledge (or conspiracy theories), and disgust at environmental degradation (or indifference) each illustrate how online technologies shape and amplify our collective emotional state.

2 Davis and Chouinard (2016) propose six ways that affordances can work. An artifact can request, demand, allow, encourage, discourage, and refuse certain actions. We can also find these mechanisms in the online environment. For instance, some features of websites are specifically designed so that they afford certain actions, like scrolling or clicking. We will return to the framework of Davis and Chouinard when we discuss affordances and social media below.

3 Here we follow Christopher Burr’s, Luciano Floridi’s, and Guy Fletcher’s definitions of digital well-being, which Fletcher describes as the “impact of digital technologies upon well-being, as opposed to some specific dimension of well-being” (2020, p. 5). For Fletcher, digital well-being extends philosophical enquiry into the nature of human flourishing by asking how digital technologies contribute to the good life in information-driven societies (Burr & Floridi 2020, p. 3; Burr et al., 2020, p. 1).

4 As Christoph Bareither (2019) explains, emotional affordances encompass the “[…] capacities to enable, prompt, and restrict the enactment of particular emotional experiences” (2019, p. 15).

5 Many theories of well-being could be described as eudaimonic, and the matter is made more complicated by the fact there are several influential definitions of well-being in circulation. See Guy Fletcher’s *The Philosophy of Well-Being* (2016) for a comprehensive account of eudaimonic or “happiness” theories, as well as a typology of other theories of well-being.

6 See Burr & Floridi for a discussion of this topic (2020, p. 6).

7 To be perfectly clear here, we are not claiming that people deliberately take the hedonic model of well-being as inspiration for a blueprint of how to design social media technology. Rather, by looking at the way social media works and its effects on people, it is *as if* the design of most social media considers the hedonic model of well-being as guide.

8 Hedonistic accounts of WB are of course part of a philosophical tradition, ranging from Aristippus of Cyrene, to Jeremy Bentham, to modern defenders like Roger Crisp. For more, see Tiberius (2014).

9 It needs to be noted that because hedonism is rather difficult to translate into quantifiable terms, psychology has adopted the concept of *subjective well-being* (Diener 1984) to better operationalize it.

10 Robert Nozick has argued as much with his thought experiments of the “experience machine” (1974). If given a choice to be either connected to a virtual reality machine that provides a (virtual) life in which you can experience a lot of pleasure, or to continue to live life with less pleasure, most people, so Nozick tells us, will prefer reality over the experience machine. However, case of the experience machine may not decisively rule out hedonism and that people may in fact have different intuitions depending on different scenarios. For more, see Stevenson (2018).

11 The complex effects of the “like” button on the Instagram platform have been catalogued by studies that show that this feature can threaten the mental health of young people. From 2019, this has led to calls to remove the “like” button. For an overview of the times that Instagram has tried to do this, see www.bbc.com/news/world-49026935 (18 July 2019).

12 To describe this phenomenon, Gilroy-Ware, borrowing from Mark Fisher (2009), uses the concept of “depressive hedonia”: To fill some void in their life, people pursue things that are supposed to give them pleasure and satisfaction but actually do not deliver on that promise.

13 Some authors use the terms “functioning well” or “optimal functioning” to describe eudaimonic WB (Keyes and Annas 2009). Although they usually overlap, there is a distinction between how people feel about their life and how they judge their functioning in life, their doing well, or their overall quality of life.

14 Eudaimonic conceptions of WB also value positive emotions because they can increase engagement in activities that facilitate agential success in realizing values. Furthermore, positive emotions are often an indirect “byproduct of eudaimonic living” (Ryan and Deci, 2001, p. 151).

15 Without going into detail here, we think that the issue of groups, collectives, and emotions could be fruitfully explored by linking it to literature on crowd psychology and the sociology of crowds. For instance, authors like Gabriel Tarde and Gustave Le Bon focused on phenomena related to crowds and interactions in groups, like imitation, contagion, and shared emotions. More recently, Tony Sampson (Sampson 2012) proposed an account of contagion that considers how processes like imitations contribute to a spread of affect and constitution of crowds in networks.

16 Peters et al. (2020) distinguish design for positive emotions and design for well-being.

17 It is more precise to say “interacting agent” here because there may be affordances for every entity that interacts with the environment, including robots.

18 There are still debates surrounding the concept of affordances, including what exactly affordances are. For more on Gibson’s view on affordances, see Jones (2003). Furthermore, affordances can enable actions but can also constrain them. To stick with our cave example, the cave affords hiding from a predator but at the same time it constrains freedom of movement. To take another example, a big and heavy rock affords smashing things but it is constraining because it is hard to lift and limits range of motion. The same thing, by virtue of the same properties, can simultaneously enable and constrain.

19 It would be misleading to say that affordances can be designed. Rather, we have to distinguish between features, affordances, and outcomes (Evans et al., 2017). Only the first one can be designed and affordances are the relations between these features and the outcome, that is what an individual does with these features. The same point is made in Anthony Chemero’s (Chemero, 2003) influential account of affordances, where affordances are characterized as relation between the abilities of the organism and the features of the environment.

20 Importantly, affordances need to be noticed or perceived in order to fulfill their purpose. If the designer wants the user to do certain things with the device then the affordances need to be marked by signifiers. For instance, the visual representation of a button on the computer screen indicates potential affordances to the user, like clickability. Some authors speak of “hidden affordances” (Gaver, 1991) when the affordance is not obvious.

21 As Thomas Jensen and Sarah Pedersen (Jensen & Pedersen, 2016, p. 84) put it: “[…] we see and experience other people’s emotions in and through their whole-body movements (facial, gestural, vocal, and postural) and likewise we enact emotions by altering our voices, moving our bodies, using our facial muscles, making gestures, or touching or disconnecting with each other.”.

22 For a review of emotional affordances, see Park & Lim (2019).

23 We aim to show how the affordances of SMTs generate emotions and emotion-related behavior, rather than how emotions affect our online behaviour.

24 We acknowledge that there is a difference between posting an emoji and actually feeling the emotion that the emoji is supposed to express. The focus here is on expressions, which could more accurately be described as signaling of emotions. In contrast to honest signaling, which guarantees reliable information and cannot be faked, emotion signaling on social media can be fake. Signaling emotions via emojis, or other digital means, can serve many functions, including semantic ones, like non-verbal cues, and purposes of meta-communication (Bai et al., 2019).

25 We thank an anonymous reviewer for bringing this point to our attention.

26 This text-centeredness cannot capture the many non-verbal aspects of communication. This negates against the expression of requisite nuances of emotions (Marin & Roeser 2020). We agree with these scholars that compared to offline communication, text-based online communication is an impoverished form of communication vis-à-vis emotions.

27 This is not to say that users do not use emojis creatively to try to approximate complex emotions. For instance, multiple emojis in a row could be used to convey complex emotional states. Besides emojis, users could use other digital means, like gifs or stickers, to express and signal more nuanced emotional states.

28 Emotional content spreads further than non-emotional content. For emotional content, it seems that negativity spreads spread more easily on social media than positivity. We do not have the space to discuss this unbalance here but would like to refer the reader to the paper by Schöne, Parkinson, and Goldenberg (2021), who found that negative language spreads further both in negative and positive situations.

29 This is also known as the bandwagon effect or heuristic, where people think something is good because others think so. See Sundar (2008).

30 For instance, Jia Tolentino (2019) reported in *The New Yorker* on so-called “Instagram face,” where users use filters to alter their face to fit a particular look.

31 The self-image of people online is closely linked to the acknowledgment of others. As Richard Seymour (2020, p. 94) put it aptly in the book *The Twittering Machine*: “Most of what there is to do in the social industry involves continually procuring a self-portrait to admire. It fuses narcissism to a digital mirror, a self-image made out of the quantified ‘reactions’ of the users.”.

32 This is not to deny that some people can grow by comparing themselves to curated versions of others on social media. However, research shows that for most people comparison on social media is associated with lower self-esteem and self-evaluation (Vogel et al., 2014).
